# In Vivo Biological Behavior of Polymer Scaffolds of Natural Origin in the Bone Repair Process

**DOI:** 10.3390/molecules26061598

**Published:** 2021-03-13

**Authors:** Fernando Bento Cunha, Karina Torres Pomini, Ana Maria de Guzzi Plepis, Virgínia da Conceição Amaro Martins, Eduardo Gomes Machado, Renato de Moraes, Marcelo de Azevedo e Souza Munhoz, Michela Vanessa Ribeiro Machado, Marco Antonio Hungaro Duarte, Murilo Priori Alcalde, Daniela Vieira Buchaim, Rogério Leone Buchaim, Victor Augusto Ramos Fernandes, Eliana de Souza Bastos Mazuqueli Pereira, André Antonio Pelegrine, Marcelo Rodrigues da Cunha

**Affiliations:** 1 Department of Morphology and Pathology, Medical College of Jundiai, Jundiaí, São Paulo 13202-550, SP, Brazil; fernandocunha21@hotmail.com (F.B.C.); emachadomd@gmail.com (E.G.M.); renatoijot@hotmail.com (R.d.M.); masmunhoz@me.com (M.d.A.eS.M.); michelanessa@hotmail.com (M.V.R.M.); victoraugustofernandes@gmail.com (V.A.R.F.); cunhamr@hotmail.com (M.R.d.C.); 2 Interunit Postgraduate Program in Bioengineering (EESC/FMRP/IQSC), University of São Paulo (USP), São Carlos 13566-590, SP, Brazil; amplepis@iqsc.usp.br; 3 Department of Biological Sciences, Bauru School of Dentistry, University of São Paulo (FOB/USP), Bauru 17012-901, SP, Brazil; karinatorrespomini@gmail.com; 4 Postgraduate Program in Structural and Functional Interactions in Rehabilitation, University of Marilia (UNIMAR), Marília 17525-902, SP, Brazil; danibuchaim@usp.br; 5 São Carlos Institute of Chemistry, University of São Paulo, USP, São Carlos 13566-590, SP, Brazil; virginia@iqsc.usp.br; 6 Department of Dentistry, Endodontics and Dental Materials, Bauru School of Dentistry, University of São Paulo (FOB/USP), Bauru 17012-901, SP, Brazil; mhungaro@fob.usp.br; 7 Department of Health Science, Unisagrado University Center, Bauru 17011-160, SP, Brazil; murilo_alcalde@hotmail.com; 8 Medical School, University Center of Adamantina (UniFAI), Adamantina 17800-000, SP, Brazil; 9 Laboratory of Anatomy, University Center Our Lady of Patronage (CEUNSP), University of South Cruise, Itu 13300-200, SP, Brazil; 10 Dentistry School, University of Marilia (UNIMAR), Marília 17525-902, SP, Brazil; elianabastosmsn@hotmail.com; 11 Research Institute, Postgraduate Program, São Leopoldo Mandic, School of Dentistry, Campinas 13045-755, SP, Brazil; pelegrineandre@gmail.com

**Keywords:** biomaterials, bone regeneration, collagen, elastin, regenerative medicine, tissue engineering

## Abstract

Autologous bone grafts, used mainly in extensive bone loss, are considered the gold standard treatment in regenerative medicine, but still have limitations mainly in relation to the amount of bone available, donor area, morbidity and creation of additional surgical area. This fact encourages tissue engineering in relation to the need to develop new biomaterials, from sources other than the individual himself. Therefore, the present study aimed to investigate the effects of an elastin and collagen matrix on the bone repair process in critical size defects in rat calvaria. The animals (Wistar rats, *n =* 30) were submitted to a surgical procedure to create the bone defect and were divided into three groups: Control Group (CG, *n =* 10), defects filled with blood clot; E24/37 Group (E24/37, *n =* 10), defects filled with bovine elastin matrix hydrolyzed for 24 h at 37 °C and C24/25 Group (C24/25, *n =* 10), defects filled with porcine collagen matrix hydrolyzed for 24 h at 25 °C. Macroscopic and radiographic analyses demonstrated the absence of inflammatory signs and infection. Microtomographical 2D and 3D images showed centripetal bone growth and restricted margins of the bone defect. Histologically, the images confirmed the pattern of bone deposition at the margins of the remaining bone and without complete closure by bone tissue. In the morphometric analysis, the groups E24/37 and C24/25 (13.68 ± 1.44; 53.20 ± 4.47, respectively) showed statistically significant differences in relation to the CG (5.86 ± 2.87). It was concluded that the matrices used as scaffolds are biocompatible and increase the formation of new bone in a critical size defect, with greater formation in the polymer derived from the intestinal serous layer of porcine origin (C24/25).

## 1. Introduction

The increasing occurrence of bone disorders such as high-energy fractures, congenital deformities, osteomyelitis, osteometabolic diseases and neoplasms that require reconstructive surgical interventions, stimulated the search for new therapies considering the limitations of natural bone regeneration in situations with marked loss of bone mass [1,2]. The autologous graft remains the gold standard in reconstructive surgical treatments, as it presents characteristics of osteoinduction, osteogenesis and osteoconduction in the regenerative process. However, it has some limitations such as limited volume of the donor area according to the surgical site, and clinical complications [3].

Tissue bioengineering has advanced in research to develop bone substitutes that meet microstructural needs, providing a favorable microenvironment for the morphofunctional restoration of original tissue [4,5]. In view of these requirements, several biomaterials have been highlighted in reconstructive surgery and regenerative medicine due to the ability to mimic tissue microstructure, allowing reactions inherent to the extracellular matrix and in the regulation of cellular phenotypes. Among these biomaterials, polymeric matrices, natural or synthetic, have shown satisfactory preclinical and clinical results [6].

Natural polymers, derived from collagen and elastin, have different shapes and densities, which simulate the native interstitial space, helping cell adhesion and supporting the proliferation and differentiation of new cell types [7,8]. Collagen is an insoluble fibrous protein considered as the main structural protein in most hard and soft tissues in animals. In bone, 90% of the extracellular matrix is collagen, which has a triple helix structure of three polypeptide chains (alpha), conferring an important role in maintaining biological and structural integrity, providing rigidity, strength and stability and providing physical support to tissues [9,10].

These properties favor the indication of collagen as a scaffold, but the mechanical properties of these products limit their applications to some extent. To overcome this deficiency, collagen scaffolds can be cross-linked by chemical or physical methods or modified with natural/synthetic polymers or inorganic materials [9,10]. Collagen matrices have the advantage of undergoing modifications in their structure when subjected to alkaline treatment, providing for the selective hydrolysis of asparagine and glutamine carboxy chains, consequently increasing negative surface charges in the molecule and piezoelectric properties. This treatment is able to transform the native matrix into a porous structure and remove remnants of an immunogenic nature [11].

Elastin-based biomaterials, on the other hand, come from the natural extraction of grafts, mainly of animal origin, consisting of aggregates of tropoelastine molecules called elastin monomers in covalent cross-linking, structuring a three-dimensional scaffold. These repeated hydrophobic sequences of elastin give the biomaterial high elasticity. Furthermore, these matrices provide characteristics necessary for tissue engineering, such as physiological degradation, biocompatibility, decreased inflammatory response and formation of new mineralized tissue [12,13].

Thus, it can be hypothesized that treatment with elastin and collagen matrices can improve the repair process of bone defects, mainly of extensive dimensions that do not close completely spontaneously, due to their physical-chemical properties and structural arrangement, which can provide an adequate scaffold for cell adhesion, differentiation and proliferation. 

However, there is no scientific research on these matrices, with the respective thermal and alkaline treatments proposed in this experimental protocol, for repairing bone defects in the craniomaxillofacial region. Therefore, the present study aimed to investigate the biological behavior of the elastin and collagen matrix in the bone repair process in a critical size defect in rat calvaria.

## 2. Results

### 2.1. Radiological and Macroscopic Analysis

The images of the macroscopic analysis were obtained on the day of euthanasia (42 days postoperatively) and the radiographic images after two days of euthanasia (44 days postoperatively), with the fixation of the pieces in 10% buffered formaldehyde (before the beginning of histological processing). 

**Figure 1:**
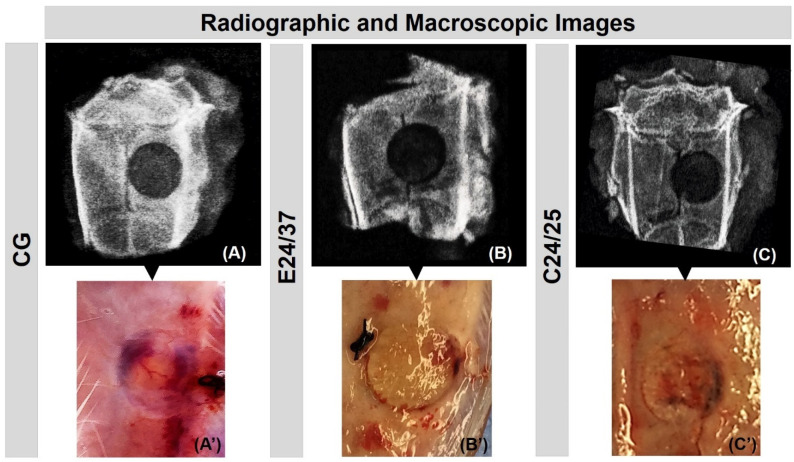
Evaluation of X-ray (**A**–**C**) and macroscopic features (**A**′–**C**′) of defects in the parietal bone rats. Groups: CG (**A**-**A**′); Hydrolyzed elastin matrix for 24 h at 37 °C—E24/37 (**B**-**B**′); Collagen matrix derived from hydrolyzed porcine intestinal serosa at 24 h at 25 °C—C24/25 (**C**-**C**′). All radiographic images show the central radiolucent cavity by removing the bone fragment in the control group (CG) and, in the groups treated with the matrices, the cavity corresponds to the low radiodensity of the biomaterials. Macroscopic images do not show any focus of infection or inflammatory process.

In the radiological analysis the absence of signs indicative of osteomyelitis or bone resorption was verified, indicating the maintenance of the conformation of the calvaria. The center of the bone defect showed a radiolucent image, indicating that there was no complete repair of the lesion (Figure 1A–C).

In the surgical area of all animals, there were no macroscopic signs of infection and, in the CG, it was possible to visualize macroscopically the presence of the blood clot in the created defect (Figure 1A′). In the biomaterial groups, E24/37 and C24/25, the presence of the matrices within the bone lesion was clearly observed. In E24/37 it was noted that the edges of the lesion were defined, allowing the identification of most of the interface with the elastin matrix (Figure 1B′), however in C24/25 it was not possible to uniformly and continuously observe the edges of the lesion and its interaction with the collagen matrix, due to the bone formation that occurred from the margins towards the center of the defect (Figure 1C′).

### 2.2. Micro CT Analysis: Three-Dimensional, Two-Dimensional Sections and Quantitative Evaluation

**Figure 2:**
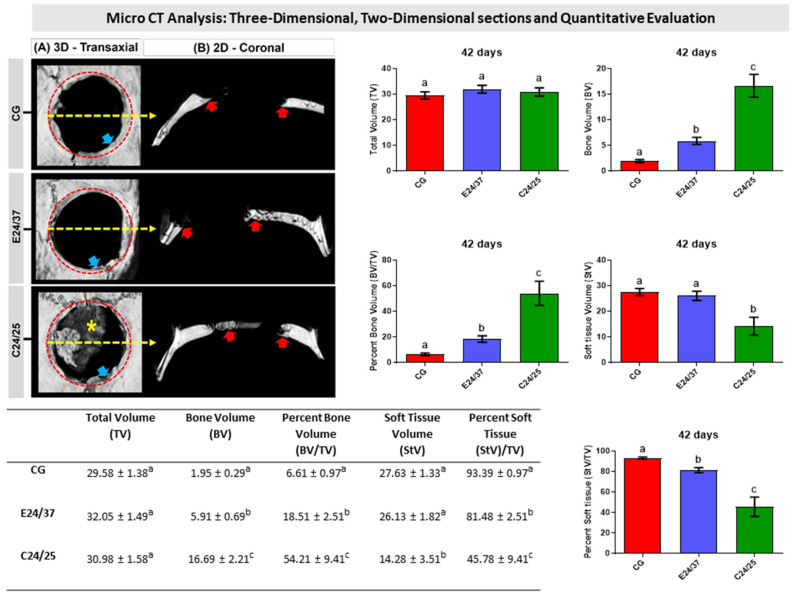
Three-dimensional and two-dimensional reconstructed micro-CT images of rat calvarial bone defects after 42 days, 3D—transaxial and 2D—coronal views of reconstructed images, (**A**,**B**, respectively). Defect without treatment (Control Group); defect filled with hydrolyzed elastin matrix for 24 h at 37 °C (E24/37 Group); defect filled with collagen matrix derived from hydrolyzed porcine intestinal serosa at 24 h at 25 °C (C24/25 Group). All groups showed a centripetal pattern of bone formation, evidenced by increased hyperdensity on the sides of the bone defect (red arrow—see dashed circle identifying the remaining bone), and thin bone plate under the dura mater in C24/25—scaffold (asterisk), remodeling areas (blue arrow) observed in margins of the remaining bone with less hyperdensity and central area of the hypodense defect. Graphs and table representing the quantitative results of the micro CT referring to Total Volume (TV, mm^3^), Bone Volume (BV, mm^3^), Percent Bone Volume (BV/TV, %), Soft tissue Volume (StV, mm^3^) and Percent Soft Tissue (StV/TV, %). In table and histogram, different lowercase letters indicate a significant difference between groups (a ≠ b ≠ c).

Forty-two days after the implantation of biomaterials the presence of elastin and collagen matrices was not detected in any of the defects treated (E24/37 and C24/25). The three-dimensional and two-dimensional images obtained by micro-CT are consistent with the histological observations, which demonstrated in all groups a centripetal pattern of bone formation, evidenced by the increased hyperdensity on the sides of the bone defect, remaining restricted in this region until the end of the experimental period (Figure 2A,B).

In the group with collagen matrix filling (C24/25), there was a greater deposition of partially mineralized bone overlapping the dura mater. All samples of this group showed areas of remodeling at the wound margins, evidenced by the gray scale in relation to the remainder. None of the groups had complete closure of the defect due to hypodensity in the central region (Figure 2A,B).

In the quantitative analysis, a significant difference was found between all groups, in the period of 42 days, in the variables bone volume (BV), percent bone volume (BV/TV) and percent soft tissue (StV/TV). There was no significant difference between all groups when evaluating the total volume (TV) of the surgically created bone defect. When measuring the soft tissue volume (StV), the C24/25 group showed a significant difference in relation to the other groups, and the CG and E24/37 groups did not present any difference between them (Figure 2).

### 2.3. Confocal Laser Scanning Microscopy Analysis

**Figure 3:**
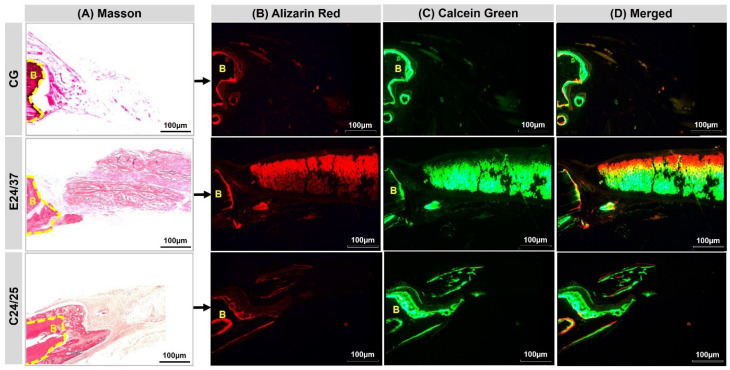
(**A**) Stained sections by Masson’s trichrome corresponding to the images observed in Confocal Laser Scanning Microscopy (CG, E24/37 and C24/25 groups). The different colors show the bone formation at different time periods. (**B**) Alizarin red applied in the immediate postoperative period and seven days after the surgical procedure. (**C**) Calcein green applied at 14 and 21 days after surgical procedure. (**D**) Overlapping images of the two fluorochromes. Remaining bone (B). Objetive ×20, Bar: 100 µm.

The images obtained in the confocal laser scanning microscopy analysis showed deposition of calcium ions at different times of bone mineralization (Figure 3). In all groups, a pattern of mineralization was observed with areas marked by fluorochrome alizarin, showing immature bone tissue close to the remaining bone and to the duramater surface, in contrast to areas marked by the green fluorochrome, calcein, corresponding to mature tissue.

Fluorescence was similar in all groups with new bone formation from the margins of the lesion, in the same areas assessed with Masson trichrome (Figure 3A). The pattern shown was the red marking of the subperiosteal young bone layer by alizarin, the first marker used, in the regions in contact with the recipient bone (Figure 3B). Calcein, shown in green, was more evident in the most central region of the lesion, showing a growth pattern starting from the remaining bone towards the center of the bone lesion (Figure 3C). In the overlapping of the images, there is a predominance of green over red, corresponding to the greater deposition of mature bone tissue (Figure 3D).

### 2.4. Mineralization Analysis of New Bone Formed by the Von Kossa’s Method

**Figure 4:**
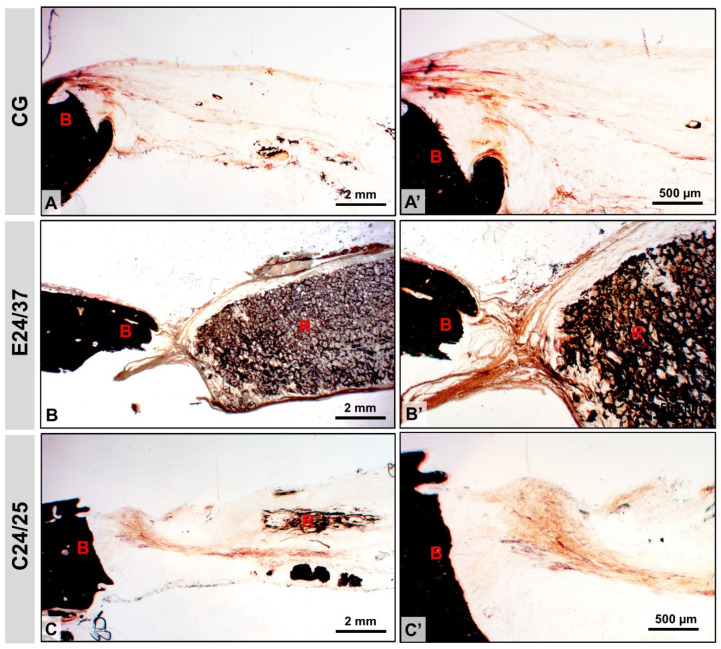
Von Kossa stained section in rat calvaria at 4× (**A**–**C**) and 10× (**A**′–**C**′) magnifications for groups: CG, E24/37, C24/25. Surgical site border (B), matrix remnant (R). Bar: 2.0 mm and 500 µm.

The CG showed the presence of fibrous connective tissue and bone trabeculae around the margins in black, indicating bone mineralization. E24/37 mineralization occurred only at the edges and, in C24/25, the presence of mineralized bone trabeculae with multiple gaps occurred more frequently in areas close to the original bone and deeply to the graft (Figure 4).

### 2.5. Birefringence Analysis of Collagen Fibers

**Figure 5:**
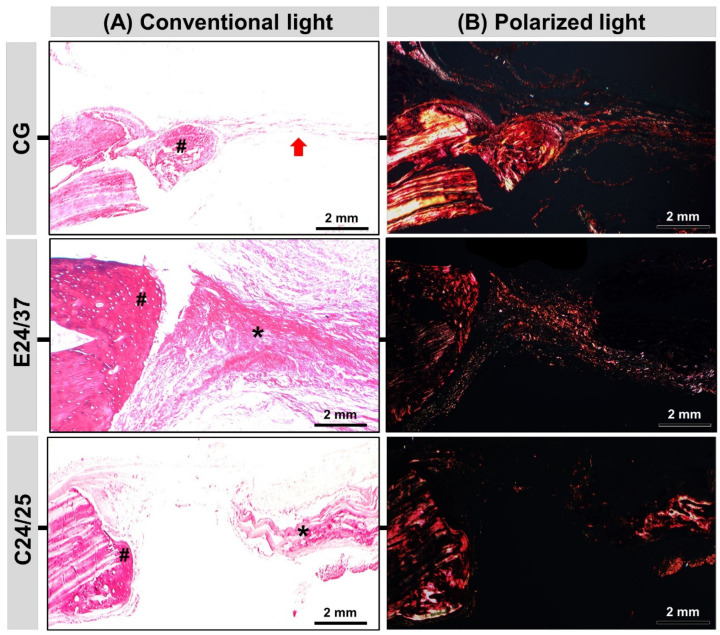
Morphological analysis of Picrosirius-hematoxylin under conventional light microscopy (**A**): presents predominance of fibrous connective tissue (red arrow) in the central region of the defect in the CG and matrices remains (asterisk) in E24/37, C24/25 during experimental period and new bone formation (hashtag) at the border of the defect. Morphological analysis of Picrosirius-polarized slices (**B**): picrosirius red stained collagen fibers are specifically birefringent in polarized light, fine green/type III fibers; thick red fibers/type I. Red-orange birefringence was observed predominantly in all groups at 42 days, corresponding to thicker and more organized collagen fibers. Note the similarity between the birefringence of the remaining collagen fibers of the newly formed tissue. Original 4× magnification, Bar: 2 mm.

Analysis of the collagen fibers stained by picrosirius red under optical and polarized microscopy allowed us to distinguish the maturation phase of the fibrils in the bone repair process in terms of thickness and compactness (Figure 5). In the polarized images, the control group (CG) showed fibers with birefringence in the transition phase in yellow-greenish areas, corresponding to type III collagen fibers (thinner and disorganized) and areas predominantly with reddish yellow birefringence, type I collagen fibers (thicker and organized). However, in the groups treated with the E24/37 and C24/25 matrices, the birefringence of the collagen fibers was prominently red (type I) similar to the remaining bone.

### 2.6. Histological Evaluation of Neoformed Bone and Histomorphometric Evaluation

**Figure 6:**
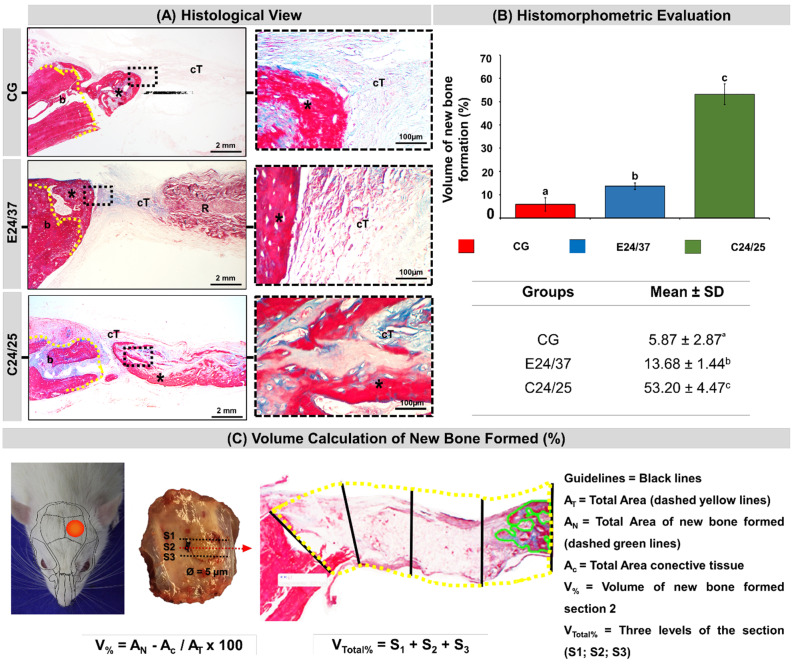
Histological images of the evolution of defects in rat calvarial in the experimental period of 42 days (**A**). Defect without treatment (CG); defect filled with hydrolyzed elastin matrix for 24 h at 37 °C (E24/37); defect filled with collagen matrix derived from hydrolyzed porcine intestinal serosa at 24 h at 25 °C (C24/25). Bone border (b), connective tissue (cT), matrix remnant (R), new bone formed (asterisk), Masson trichrome. Original 10× magnification; bar = 500 µm and inserts, enlarged 40× images; bar = 100 µm. Histomorphometric Evaluation: graph of the volume of new bone formed (%) in each group at 42 days. Table of percentages of new bone formed from all groups: (**B**). Analysis was performed between the different groups (CG, E24/37 and C24/25). ANOVA a criterion followed by the Tukey’s test. Mean ± standard deviation (*n* = 10 animals/group), where different letters (a ≠ b ≠ c) indicate statistically significant differences (*p* < 0.05). (**B**). Ilustration in the volume calculation of new bone formed (%) of all groups (**C**).

In all experimental groups at 42 days after surgery, a centripetal pattern of new bone formation was observed, projecting from the margins to the center of the defect. Bone regeneration was partial with the presence of new bone tissue formed restricted to the periphery and, in the central area, it was inserted by fibrous connective tissue (Figure 6A).

The defects treated with the matrices did not show signs of necrosis or chronic inflammatory infiltrates. The new bone tissue formed was shown to be mature, but without restoring the height of the native bone. At 42 days, there were remnants of the elastin and collagen matrices in the center of the surgical defect (Figure 6A).

In the quantitative evaluation of the relative percentage volume of newly formed bone, it was observed that defects treated with matrices, C24/25 and E24/37 (53.20 ± 4.47 and 13.68 ± 1.44, respectively) had higher means with significant difference compared to the control group (5.87 ± 2.87) (Figure 6B).

## 3. Discussion

Advances in the area of tissue engineering have enabled the development and improvement of new biomaterials to provide the recovery of the functional and morphological capacity of the injured tissues. In the search for improving the characteristics of scaffolding in recent years, alternatives have been studied with greater intensity [14,15]. Therefore, this experimental protocol investigated the effects of elastin and collagen matrices on cranial defects in rats and, according to the results, it is possible to state that the collagen matrix was more effective in the regenerative process. The research focused on the in vivo analysis of two polymer scaffolds for biomedical applications compared to a group without polymers, according to the special issue of the scientific journal Molecules.

Biocompatibility is one of the characteristics to be analyzed in the process of using the new biomaterials and is related to the ability of a material to generate an adequate response in the receiving bed with the absence or minimal possibility of inflammatory, toxic or mutagenic reactions [16,17]. The polymeric membranes used in this experiment, derived from bovine and porcine tissue, demonstrated this characteristic mainly due to the alkaline hydrolysis process that completely removed the native tissue cells [7,18,19].

The alkaline treatment of the polymers, in addition to promoting the acellularization that could cause immunological reactions, transforms the matrices into porous structures, which favor the migration and adhesion of precursor cells from new tissue. In this study, the elastin membrane treated in alkaline hydrolysis for 24 h at 37 °C and the collagen membrane for 24 h at 25 °C, provided the absence of inflammatory and infectious signs of tissue rejection, both macroscopic and histological, in the groups grafted with the biomaterials, proving to be nontoxic and viable for later clinical application [20,21,22]. 

In the radiological analysis, the absence of signs indicating complications in the repair of the defect and in the morphological maintenance of the calvaria, is one of the indications of compatibility of the implanted matrices with the host tissue. Studies using scaffolds have in common the purpose of providing an appropriate microenvironment for cells to migrate and proliferate, differentiating themselves in the new tissue, such as hydrogels [23], fibrin sealants [24], bone grafts [25] and other methods that use biomaterials in regenerative medicine. 

Microtomographically, the presence of elastin and collagen matrices was not detected in any of the defects treated (E24/37 and C24/25), probably due to the process of biodegradation with a concomitant rate of bone neoformation, being an essential factor as it promotes cell proliferation and, at the same time, allows space for bone growth [26]. Centripetal formation was still be observed, more evident on C24/25, which showed a greater deposition of partially mineralized bone, a fact associated with the porous intestinal serosa membrane, abundant in collagen, and a three-dimensional biomaterial with cellular affinity and elasticity, which allows the creation of a microenvironment facilitating cell growth [11,19,27,28].

In the microscopic confocal analysis, immature bone was found close to the defect margins and to the duramater, and marked with calcein in the central region. Type I collagen fibers in groups E24/37 and C24/25, seen in birefringence analysis, were found in grafts that used matrices that had undergone alkaline hydrolysis, increasing the number of negative surface charges in the molecule and increasing the fundamental piezoelectric properties for osteogenesis [29,30,31].

Microscopically, in the performed stains (Masson’s trichrome and Von Kossa), at 42 days, in the CG the neoformation was restricted to the periphery and in the treated groups, especially in C24/25, the new bone was mature and also present in the central region of the defect. The porcine intestinal collagen membrane showed better osteoregenerative capacity compared to elastin. This may be related to its greater roughness and better hydrophilic/hydrophobic balance, as observed in previous studies [32,33,34].

In the quantitative analysis, in the mean values of new bone formation the elastin membrane (E24/37) provided a 134% increase in bone volume in relation to the control group (CG = 5.86 ± 2.87; E24/37 = 13.68 ± 1.44), and the porcine collagen membrane 817% in relation to the CG (C24/25 = 53.20 ± 4.47). Previous studies using the collagen membrane derived from bovine intestinal submucosa obtained satisfactory results in terms of osteogenesis and angiogenesis [35,36]. However, there are few studies with the membrane derived from the serous intestine layer porcine slender, used in this experimental protocol, that proved to be a potential scaffold for bone grafts in regenerative medicine and tissue engineering.

The porcine intestinal collagen membrane demonstrated greater osteoregenerative capacity compared to elastin. This may be related to its greater roughness, low immunogenicity, a porous structure, better hydrophilic/hydrophobic balance and, consequently, greater degradation speed concomitant to the bone neoformation process [32,33,34]. The scaffolds produced from collagen have the potential for a better regeneration result due to their similarity with the composition of the native bone matrix and physicochemical properties that can promote an adequate biological response, such as cellular interaction with the ability to function as extracellular biomimetic matrices that guide tissue regeneration [9,10]. 

However, elastin membranes can also be considered as promising products for clinical applications since the morphometric data demonstrated that there was greater bone formation in the surgical area when compared to the control. The results obtained in this experimental protocol regarding the parameter of new bone formation allow the future translation of this preclinical study to clinical procedures. Previous studies of other biomaterials sought to resolve similar situations; for example, the application of intrafibrillar silicified collagen scaffold in bone, which demonstrated the promoted angiogenesis and osteogenesis responded to the reaction between the degrading scaffold and monocytes recruited to the defect [37].

In the last decade, studies with structured hydrogels (multifunctional 3D architectured hydrogels) have increased, the hydrogels showing shape stability and elastic properties that are individually adjustable in different length scales and produced in one step. They have demonstrated cell adhesion and differentiation capabilities and exhibit increasing pore size during degradation [38]. Lohmann et al. [39] analyzed the healing potential of porous ArcGel compared to autologous bone (gold standard) and Bio-Oss^®^ Collagen (Bio-Oss), a last generation bone graft biomaterial and frequently used clinically. They found promising results because the amount of bone formed after the implantation of ArcGel was comparable to autologous bone and superior to Bio-Oss^®^. The microarchitecture of newly formed bone was more physiological and better functional in the case of ArcGel.

These findings lead to prospective studies, with new materials and clinical tests, aiming at the morphological and functional rehabilitation of extensive bone defects. 

## 4. Materials and Methods 

### 4.1. Elastin Matrix Derived from Bovine Auricular Cartilage

**Figure 7:**
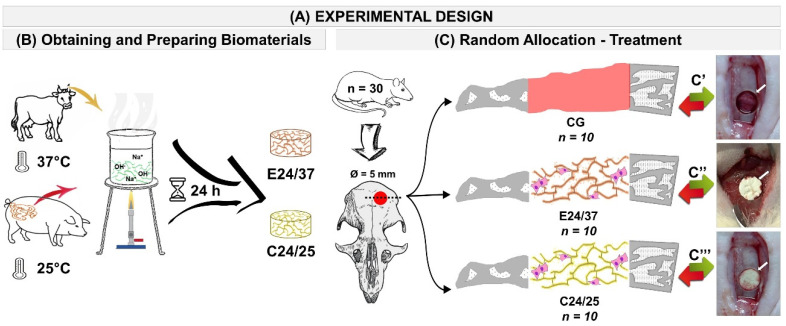
Schematic drawing of the experimental design (**A**). Preparation and application of biomaterials. Hydrolysis bovine auricular cartilage at 37 °C for 24 h and hydrolysis porcine intestinal serosa 24 h at 25 °C (**B**). Random Allocation—Treatment: Inclusion criteria: 30 male rats, *Rattus norvegicus*, Wistar, 112 days old and average weight of 360 g were randomly separated into three groups: Control Group (CG, *n* = 10), calvarial defects filled with blood clot (without scaffold); E24/37 (*n* = 10), bovine elastin matrix filled calvarial defects hydrolysed for 24 h at 37 °C and C24/25 (*n* = 10), porcine collagen matrix filled calvarial defects hydrolyzed for 24 h at 25 °C (**C**). Surgical procedure: **C**′—bone defect without scaffold; **C**″—bone defect filled E24/37 and **C′′′**—bone defect filled C24/25.

The manufacturing process of elastin and collagen matrices and their characterizations have been described in greater detail in previously published studies [7,19,27,40,41,42]. The elastin matrices used were prepared by the Biochemistry and Biomaterials Group of the São Carlos Institute of Chemistry (University of São Paulo—USP, Brazil) by adapting the methodology used to obtain collagen [7,8,14]. In summary, the cartilages were treated with an alkaline solution, a mechanism known as hydrolysis, with salts sulfates and chlorides and alkaline earth alkali metal hydroxides at 37 °C for 24 h (E24/37). After the hydrolysis period, the resulting materials were equilibrated in a solution containing chlorine and alkaline sulfate (potassium, calcium and sodium). Excess salts were removed by washing in 3% (*w*/*w*) boric acid solution, deionized water, followed by 0.3% (*w*/*w*) EDTA solution, and finally in deionized water. The resulting material was equilibrated at pH 7.4 in saline phosphate buffer for 24 h and thoroughly washed with deionized water, thenfrozen, lyophilized and kept in aseptic containers. Prior to implantation, the elastin matrices were cut into discs 1 mm thick × Ø 5 mm and hydrated in sterile saline for 24 h (Figure 7A,B).

### 4.2. Collagen Matrices Derived from Porcine Intestinal Serosa

The collagen matrices used were prepared by the Biochemistry and Biomaterials Group of the São Carlos Institute of Chemistry—USP. After obtaining the porcine intestinal serosa, washing in 0.9% saline (NaCl) and distilled water began. The material was divided into two portions, native matrix and anionic matrix. After cleaning, for the preparation of the anionic matrix, the material was initially placed in an alkaline solution for 24 h at a temperature of 25 °C (C24/25). Subsequently, the treatment steps followed the same protocol to obtain the elastin matrix derived from bovine auricular cartilage. Prior to implantation, the collagen matrices were cut into discs 1 mm thick × Ø 5 mm and hydrated in sterile saline for 24 h (Figure 7A,B).

### 4.3. Experimental Design

Thirty male rats, *Rattus norvegicus*, Wistar, 112 days old and average weight of 360 g were used. The animals were supplied by the Institute of Energy and Nuclear Research (São Paulo—SP) and kept in the bioterium of the Jundiaí Medical School (FMJ). All animals were separated into boxes with a maximum of three animals each, and received balanced feed (Purina™, SP, Brazil) and water ad libitum. The environment was maintained with controlled temperature (23 ± 1 °C) and a light and dark cycle every 12 h.

All experimental animal procedures were conducted with the approval of the Animal Research Ethics Committee of the Jundiai Medical School (CEUA/FMJ), protocol 56/2015.

The animals were randomly divided into three groups: Control Group (CG, *n =* 10), calvaria defect filled with blood clot (without scaffold); E24/37 Group (*n =* 10), bovine elastin matrix filled calvaria defect hydrolysed for 24 h at 37 °C, and C24/25 Group (*n =* 10), porcine collagen matrix filled calvaria defect hydrolyzed for 24 h at 25 °C (Figure 7A–C).

### 4.4. Bone Defect Surgeries

Animal surgeries were performed under dissociative anesthesia by gluteal intramuscular (IM) injection with ketamine (50 mg/kg im, Dopalen™, Ceva, SP, Brazil), and xylazine (10 mg/kg im, Coopazine™, Coopers, SP, Brazil), at a ratio of 1:1 (0.10 mg/100 g body mass) and 5 min before the surgical procedure, tramadol hydrochloride 50 mg/mL IP (5 mg/kg body mass, Cronidor™, Agener União, SP, Brazil) was applied as an analgesia. Rats were positioned on the operation table in ventral decubitus. 

Asepsis and trichotomy of the calvaria and adjacent areas were performed. The skin was incised longitudinally and folded together with the periosteum. With the use of a trephine drill (Härte Precision Grip^TM^, São Paulo, Brazil) at low speed coupled to the Beltec^TM^ LB100 micromotor (Beltec Micromotors, SP, Brazil) a bone defect (Ø = 5mm) was created in the left parietal, with constant saline irrigation to avoid thermal necrosis, and 0.9% saline solution was used to remove residues. 

The bone grafts were inserted into the defects created surgically according to the allocation of the groups, except for the CG, which remained filled by clot. Then, the periosteum and the skin were sutured separately.

After surgery, a single IM injection of pentabiotic at a dose of 0.1 mg/100 g IM (Fort Dodge^TM^, SP, Brazil), topical sodium rifamycin (Rifocina^TM^, Sanofi-Aventis Pharmaceutical Ltd.a, SP, Brazil) was administered and oral administration acetaminophen at a dose of 200 mg/kg (Paracetamol^TM^, Medley, São Paulo, Brazil) dissolved in water was available in drinking fountains for 14 days.

Throughout the experimentation, the animals were monitored for expression of pain by observing whether the animal was apathetic, depressed, aggressive or overexcited, such traits being variable in their usual behavior. Changes in walking, posture or facial expression were also observed and the appearance, water consumption, food and clinical symptoms were investigated. There were no complications that needed to be reported, and there was no disease or sign that strongly motivated the removal of an animal (clinical outcome).

The animals were euthanized 42 days after the surgical procedure with excessive dose of xylazine and ketamine, followed by pneumothorax induced by sectioning of the diaphragm through the abdominal cavity. Then, the calvaria was resected with the use of a Taimim^TM^ oscillatory saw (model YDJZ-IID; Shanghai Xinsheng Photoelectric Technology CO., Ltd., Shanghai, China).

The samples were separated according to the staining to be performed, the state of calcification and the inclusion medium. Five animals per group followed the protocol for laser scanning confocal microscopy and Von Kossa method, and five animals per group for macroscopic, radiological, micro CT, Masson trichrome and picrosirius red staining.

### 4.5. Sequential Fluorescent Labeling

Five animals from each experimental group underwent dorsal subcutaneous administration of fluorochromes in the postoperative period. Alizarin Red S 30 mg/kg injections (Sigma-Aldrich^TM^, Merck KGaA, Darmstadt, Germany) were performed in the immediate postoperative period and seven days after the surgical procedure. Calcein 10 mg/kg (Sigma-Aldrich^TM^, Merck KGaA, Darmstadt, Germany) injections were performed at 14 and 21 days after the surgical procedure. All dyes were prepared immediately before use with disodium phosphate and saline solution.

### 4.6. Histologic Preparation for Confocal Laser Scanning Microscopy Analysis

The samples were fixed in 10% buffered formaldehyde for two days and sequentially dehydrated in increasing concentrations of 60 to 100% alcohols, embedded in glycol methacrylate resin and polymerized (4 h under white light and 96 h under blue light). After complete polymerization, coronal and semiserial histological sections of 200 μm thickness were made and subsequently reduced with the aid of an automatic sander (2300 rpm speed) to 30–50 μm by the Exakt Cutting Grinding system (Exakt^TM^Apparatus GmbH, Norderstedt, Germany).

The sections were analyzed using a TCS SP5AOBS laser scanning confocal microscope (Leica^TM^, Wetzlar, Germany), coupled with a DFC 310 FX camera (Leica^TM^, Wetzlar, Germany), and QWin 3.1 (Leica^TM^, Wetzlar, Germany). The photomultiplier for each of the fluorescence markers was 488 nm (calcein) and 543 nm (alizarin red).

### 4.7. Histologic Preparation and Von Kossa’s Staining Method

The preparation of slides for the Von Kossa staining method, to verify extracellular matrix mineralization such as calcium and potassium ions in newly formed bone tissue, followed the same protocol as the confocal laser scanning microscopy analysis. The slides were washed three times in distilled water and immersed in 1% silver nitrate solution under ultraviolet radiation for 1 h. After this process, they were immersed in 5% sodium thiosulfate solution and immediately washed in distilled water. This process produced pink and black colorations, indicating the cytoplasm and calcium ions, respectively. Cell nuclei were counterstained by immersion for 5 min in safranin solution and glacial acetic acid. The slides were observed under a Motic BA310E series optical microscope (Motic^TM^, Kowloon, Hong Kong) and images captured on 4× and 10× lenses.

### 4.8. Macroscopic and Radiological Analysis

The removed calvaria was documented with a Nikon^TM^ digital camera model D3500 DSLR (Tokyo, Japan), assessing the presence of any pathological changes or any other abnormal condition in the surgical area. Then, samples were radiographed with an Odel 300 mA, 100 mA focus, time of 0.06 s and 40 kV radiation and digitalized by the Agfa^TM^ system (Japan) to assess bone defect integrity, adjacent areas and bone repair.

### 4.9. Micro-CT Scan (μ-CT)

The samples were subjected to an X-ray beam scan in a SkyScan 1174v2 computed micrographograph machine (μ-CT-Bruker-microCT ^TM^, Kontich, Belgium). The X-ray system is based on a micro focus tube (50 kV, 800 μA) generating projection images irradiating X-rays with cone beam geometry. The calvaria were placed on a computer-controlled rotation platform and scanned 180° around the vertical axis in rotation steps of 0.73° at 50 kV. The images were captured with 16.61 µm and further reconstructed using the NRecon^TM^ v.1.6.8.0 program (SkyScan ^TM^, 2011, Bruker-microCT), with the same reconstruction parameters for all specimens. Next, the reconstructed images were realigned using the DataViewer ^TM^ 1.4.4.0 software resulting in two-dimensional transaxial and sagittal images with 16 bits grey scale resolution. Next, images were reconstructed three-dimensionally using CTvox.

The region of interest (ROI) was chosen as a sleeve around neoformed bone (NFB) and bone particles individually in the coronal plan, using the CTAn^TM^ 1.10.1.0 software (SkyScan^TM^, Belgium). Subsequently, the binarization of the images was performed with the upper and lower limits (grayscale threshold) for NFB 130-22, with number of layers 207, then the calculation of the respective volumes. Afterwards, total defect volume (TV), the bone volume (BV) and percent bone volume (BV/TV) were measured for each dataset, and soft tissue volume (StV) was measured by subtracting the defect volume (TV) from the bone volume (BV). Soft tissue percent (StV)/TV) was measured by dividing the soft tissue volume (StV) by defect volume (TV).

### 4.10. Histological Procedures–Masson’s Trichrome Staining and Picrosirius Red

After micro-CT scanning, calvaria samples were decalcified in solution for descaling hydrogen chloride Allkimia^TM^ (Allkimia Trade Materials Laboratory Ltd., SP, Brazil) for 20 days. After this period, the acid action was neutralized for 24 h in a 5% sodium sulfate solution. Subsequently, the samples were histologically processed for embedding in Histosec^TM^ (paraffin enriched with polymers of the EMD Millipore—division of Merck KGaA, Darmstadt, Germany). Semiserial coronal sections of 5 μm thick (three different points of bone failure—1250 μm intervals) of all defects were performed, collected on slides and stained with Masson’s trichrome and picrosirius red. The slides were observed under light microscope and polarized light for determining the quality of the newly formed organic matrix. The histological analysis of the Masson’s trichrome stained sections consisted of the evaluative description of the bone repair process for each treatment. Motic BA310E series microscope (Motic^TM^, Kowloon, Hong Kong) was used and digital images were analyzed with 4× and 40× objectives.

### 4.11. Histomorphometric Evaluation

Masson’s Trichrome staining was used because it allows the differentiation between the remaining bone (mature-cortical) staining in red from the newly formed bone (immature-trabecular) staining in blue. Objective digital images (4×) were used to quantify newly formed bone volume using the Motic Images Plus 2.0 software (Motic Digital Microscopy^TM^, Kowloon, Hong Kong). Bone defect margins and cortical thickness were marked with a black guideline throughout the bone defect to maintain the original bone outline of each animal (the guidelines had the same measurements as the cortical thickness). 

A yellow line was manually drawn around all the bone defects, having as reference the limits of the guidelines, to obtain the total area of the bone failure. The areas of new bone formation were marked with a green line. Thus, the data relating to the total area of bone defect (AT), total area of neoformation (AN) and the area of connective tissue (AC), the percentage of bone regeneration of each histological section, were obtained through the formula: V(%) = An − Ac/At × 100 (Figure 6C).

### 4.12. Statistical Analysis

Data regarding the percentage of bone neoformation volume of the surgical area of each rat (histomorphometric analysis), as well as the measurements of the micro-CT, were transcribed to the GraphPad Prism version 8.0.1, applying ANOVA tests followed by the Tukey‘s test for statistical evaluation and obtaining means and standard deviations between the study groups with a significance level set at *p* < 0.05. The homogeneity of variances and normality were tested by the Shapiro–Wilk and Bartlett tests, both at 5% probability.

## 5. Conclusions

The biological behavior of two scaffolds (matrices derived from elastin and collagen) in critical bone defects of rat calvaria were evaluated in this study in vivo. The results showed that the two three-dimensional matrices were biocompatible and increased the formation of new bone in relation to the control group (CG), in which the surgical bed was filled only by a blood clot, with a greater quantitative response of the collagen matrix derived from the intestinal serous layer of porcine origin (C24/25 Group). Due to the favorable biological response in the host tissue, ease in obtaining native material (donor sources), available quantity and low production cost, there is translational potential of the biomaterials evaluated, with prospects for use in clinical protocols.

## Data Availability

The data presented in this study are available on request from the corresponding author. The data are not publicly available due to they are part of a master’s dissertation not yet deposited in a public repository.

## Sample Availability

Samples of the elastin and collagen matrices are available from the authors.
